# Preferential killing of multidrug-resistant KB cells by inhibitors of glucosylceramide synthase

**DOI:** 10.1038/sj.bjc.6690711

**Published:** 1999-10

**Authors:** K M Nicholson, D M Quinn, G L Kellett, J R Warr

**Affiliations:** Department of Biology, University of York, PO Box 373, York, Y010 5YW, UK

**Keywords:** multidrug-resistance, glucosylceramide, apoptosis

## Abstract

This study has compared the preferential killing of three multidrug-resistant (MDR) KB cell lines, KB-C1, KB-A1 and KB-V1 by two inhibitors of glucosylceramide synthase, 1-phenyl-2-decanoylamino-3-morpholino-1-propanol (PDMP) and 1-phenyl-2-hexadecanoylamino-3-pyrrolidino-1-propanol (PPPP), to the killing produced by these compounds in the drug-sensitive cell line, KB-3-1. Both of the inhibitors caused much greater induction of apoptosis in each of the three MDR cell lines than in the drug-sensitive cell line, as judged by morphological assay and confirmed by poly-(ADP-ribose)-polymerase cleavage. The highest level of apoptosis was produced following 24-h exposure to 5 μM PPPP. This treatment produced 75.8 (± 7.1)%, 73.6 (± 9.8)% and 75.3 (± 6.4)% apoptotic cells in the three MDR cell lines respectively, compared to 19.0 (± 9.8)% in the drug-sensitive cell line. A reduction in glucosylceramide level following inhibitor treatment occurred in KB-3-1 cells as well as in the MDR cell lines, suggesting that the increased apoptotic response in the MDR cells reflected a different downstream response to changes in the levels of this lipid in these cells compared to that in the drug-sensitive cells. These results suggest that the manipulation of glucosylceramide levels may be a fruitful way of causing the preferential killing of MDR cells in vitro and possibly in vivo. © 1999 Cancer Research Campaign


*British Journal of Cancer* (1999) **81**(3), 423–430
© 1999 Cancer Research Campaign
Article no. bjoc.1999.0711

### Preferential killing of multidrug-resistant KB cells by inhibitors of glucosylceramide synthase

**KM Nicholson, DM Quinn, GL Kellett and JR Warr**

Department of Biology, University of York, PO Box 373, York Y010 5YW, UK

**Summary** This study has compared the preferential killing of three multidrug-resistant (MDR) KB cell lines, KB-C1, KB-A1 and KB-V1 by two inhibitors of glucosylceramide synthase, 1-phenyl-2-decanoylamino-3-morpholino-1-propanol (PDMP) and 1-phenyl-2-hexadecanoylamino-3-pyrrolidino-1-propanol (PPPP), to the killing produced by these compounds in the drug-sensitive cell line, KB-3-1. Both of the inhibitors caused much greater induction of apoptosis in each of the three MDR cell lines than in the drug-sensitive cell line, as judged by morphological assay and confirmed by poly-(ADP-ribose)-polymerase cleavage. The highest level of apoptosis was produced following 24-h exposure to 5 μM PPPP. This treatment produced 75.8 (± 7.1)%, 73.6 (± 9.8)% and 75.3 (± 6.4)% apoptotic cells in the three MDR cell lines respectively, compared to 19.0 (± 9.8)% in the drug-sensitive cell line. A reduction in glucosylceramide level following inhibitor treatment occurred in KB-3-1 cells as well as in the MDR cell lines, suggesting that the increased apoptotic response in the MDR cells reflected a different downstream response to changes in the levels of this lipid in these cells compared to that in the drug-sensitive cells. These results suggest that the manipulation of glucosylceramide levels may be a fruitful way of causing the preferential killing of MDR cells in vitro and possibly in vivo.
© 1999 Cancer Research Campaign

**Keywords:** multidrug-resistance; glucosylceramide; apoptosis

Many factors have been linked to the failure of chemotherapy as an effective treatment for cancer. One of the major obstacles to the success of chemotherapy is drug resistance at the cellular level. Drug resistance can be innate or acquired and often becomes apparent on exposure to a single cytotoxic agent, which selects cells resistant to other functionally and structurally unrelated drugs (reviewed by Ling, 1992). This phenomenon is termed multidrug resistance and is commonly associated with overexpression of the 170 kDa ATP-dependent drug efflux pump termed P-glycoprotein (P-gp), encoded by the *MDR1* gene in humans. P-glycoprotein reduces the intracellular concentration of drug by actively increasing cellular drug efflux. In light of this, the approach most extensively employed in an attempt to circumvent multidrug resistance has involved the use of resistance modifiers such as verapamil to reverse P-gp function. However, so far this approach has had limited clinical impact (reviewed by Volm, 1998).

Other changes have been reported in multidrug-resistant (MDR) cells in addition to P-gp overexpression. These changes suggest an alternative approach to circumvent multidrug resistance by exploiting these biochemical differences to eradicate MDR cells preferentially. One such difference involves changes in metabolism, such as the increased levels of glycolysis shown by some MDR cells (Cohen and Lyon, 1987). Previous results from this laboratory have shown that MDR variants of the KB carcinoma cell line were preferentially sensitive to the effects of the glycolytic inhibitor 2-deoxy-D-glucose (Bentley et al, 1996) and to tunicamycin (Bentley et al, 1997), which in the former case was shown to be via an induction of apoptosis (Bell et al, 1998).

*Received 26 February 1999*
*Revised 19 May 1999*
*Accepted 21 May 1999*

*Correspondence to:* JR Warr

A recent report has suggested that accumulation of the glycosphingolipid glucosylceramide is a feature of MDR cells, which may be a requirement for the acquisition and/or maintenance of multidrug resistance (Lavie et al, 1996). This phenomenon has also recently been observed in a limited study in patients with melanoma (stage IV) and breast cancer (stage IV), where measurable levels of glucosylceramide were detected in tumour specimens from patients who failed chemotherapy, but not in those from patients with a clinical response following chemotherapy (Lucci et al, 1998).

Glucosylceramide is formed from ceramide, by the action of the enzyme glucosylceramide synthase, which mediates the transfer of UDP-glucose to ceramide. Glucosylceramide is further glycosylated to other higher gangliosides and glycolipids or is degraded by glucocerebrosidase (Marsh et al, 1995). Ceramide, the precursor of glucosylceramide, is recognized as a second messenger involved in the induction of apoptosis, although this view has recently been challenged (Hoffman and Dixit, 1998). Generation of ceramide through the sphingomyelin-signalling pathway occurs in response to the post-receptor action of a variety of cytokines, hormones and growth factors (Jarvis et al, 1996). These include members of the tumour necrosis factor (TNF) superfamily, Fas/Apo-1 ligand, interleukin-1 and 1,25-dihydroxyvitamin $D_3$. On activation of the corresponding receptors, sphingomyelin is hydrolysed to ceramide and phosphorylcholine by the action of a sphingomyelinase. Alternatively, de novo synthesis of ceramide occurs from sphingosine mediated by the enzyme ceramide synthase (Luberto et al, 1998). Among the intracellular targets of ceramide are a ceramide-activated protein kinase (CAPK; Mathias et al, 1991), ceramide-activated protein phosphatase (CAPP; Dobrowsky and Hannun, 1992) and protein kinase Cξ (Lozano et al, 1994).

Inhibitors of glucosylceramide synthase have been well-documented and include PDMP (1-phenyl-2-decanoylamino-3-morpholino-1-propanol), (Figure 1), which causes the accumulation

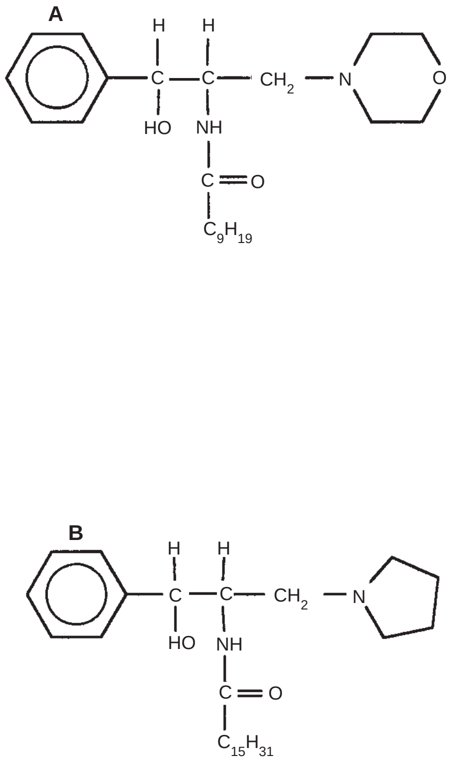

**Figure 1** The structure of (**A**) PDMP and (**B**) PPPP

of ceramide and a reduction of glucosylceramide (Radin et al, 1993), and PPPP (1-phenyl-2-hexadecanoylamino-3-pyrrolidino-1-propanol), (Figure 1), which has been reported to only cause a reduction in glucosylceramide without affecting ceramide levels (Abe et al, 1995). PDMP was synthesized as a specific treatment for Gaucher's disease in which patients have increased levels of glucosylceramide due to a lack of glucocerebrosidase (Radin, 1996). In addition, preliminary studies designed to assess the efficacy of PDMP as an anticancer agent demonstrated that mice inoculated with Ehrlich ascites carcinoma cells and treated with PDMP had an increased lifespan in relation to control mice (Radin, 1994). In another study using rats with C6 glial tumours, PDMP reduced tumour size (Radin, 1994). PPPP was rationally designed based on the structure of PDMP, in which the morpholine group in PDMP has been substituted by a pyrrolidine group to increase the specificity and efficacy of PPPP as an inhibitor of glucosylceramide synthase (Abe et al, 1995).

This present study was designed to investigate the effect of inhibitors of ceramide metabolism on MDR cells in view of the evidence of increased glucosylceramide accumulation in MDR cells. An additional aim was to determine the nature of any preferential cell killing effect of these inhibitors in terms of induction of apoptosis or necrosis, as understanding the mechanism of killing of MDR cells by these agents will be of value in optimizing the process. From this study it is apparent that altering ceramide metabolism with PDMP and PPPP has a preferential killing effect on MDR KB cells. These findings suggest that the maintenance of glucosylceramide levels may be of greater significance in preventing apoptosis in MDR cells than in their normal counterparts.

### MATERIALS AND METHODS

### Cell culture

The human KB carcinoma drug-sensitive cell line, KB-3-1, and its MDR derivative cells, KB-C1, KB-A1 and KB-V1, selected in colchicine, doxorubicin and vinblastine respectively, were obtained from Dr MM Gottesman (National Cancer Institute, Bethesda, MD, USA) or the American Type Culture Collection. Cells were maintained in 25-cm$^2$ flasks in Dulbecco's modified Eagle's medium (DMEM; Gibco) containing 0.11 g $l^{-1}$ sodium pyruvate and 4.5 g $l^{-1}$ glucose, supplemented with 10% fetal calf serum (v/v), penicillin (32 μg $ml^{-1}$) and streptomycin (50 μg $ml^{-1}$). The MDR variants of the KB cell lines were maintained in the presence of 1 μg $ml^{-1}$ of the selecting agent. Cells were grown as monolayers and were incubated at 37°C in a humidified atmosphere of 95% air/5% carbon dioxide. Cells were grown in the absence of selecting agent during each experiment.

DL-*threo*-PDMP (Calbiochem) was dissolved in isopropanol to a concentration of 50 mM and was further diluted to working concentrations by the addition of DMEM. DL-*threo*-PPPP (Calbiochem) was dissolved in dimethyl sulphoxide to a concentration of 100 mM and was further diluted to working concentrations by the addition of DMEM. The final concentration of solvents used was not found to be cytotoxic to the cells.

### Colony formation assay

KB-3-1 and its MDR derivatives were incubated in 6-well plates in the presence of PDMP or PPPP for 24 h. Cell survival was measured by a colony-forming assay, in which cells were re-plated at a density of between 100 and 300 cells per well in 24-well plates, depending on the plating efficiency of the cell line, for a further 8 days before staining with Leishman's stain (0.2% in methanol). Colonies of over 50 cells were counted, and survival was expressed as colony forming ability after treatment with inhibitor relative to that of untreated controls.

### Morphological assessment of apoptosis induction in cells exposed to PDMP and PPPP

Cells were plated at an initial cell density of $4 \times 10^4$ cells per well in 6-well plates for 2 days. DMEM was removed and replaced with PPPP or PDMP. Following a specific exposure time all cells (detached and adherent) were harvested and were labelled with annexin-V–FITC (fluoroscein isothiocyanate; final concentration 4 μg $ml^{-1}$) in annexin binding buffer (10 mM HEPES pH 7.4, 150 mM sodium chloride, 5 mM potassium chloride, 1 mM magnesium chloride and 1.8 mM calcium chloride), Hoechst 33342 (final concentration 16 μg $ml^{-1}$) and propidium iodide (final concentration 10 μg $ml^{-1}$). Cells were viewed under a fluorescence microscope (Zeiss) using a UV filter (365 nm) for viewing Hoechst 33342 and propidium iodide staining and a blue filter (450–490 nm) for annexin-V–FITC and propidium iodide staining. Three hundred cells per sample were counted in triplicate for each time point. Cells were scored as viable, apoptotic and necrotic as judged by nuclear morphology, membrane integrity and phosphatidylserine externalization. Viable cells were blue and the nucleus was diffuse and intact. Apoptotic cells were divided into early and late apoptotic; early apoptotic cells were blue since they maintain their membrane integrity and exclude propidium iodide, however

© 1999 Cancer Research Campaign

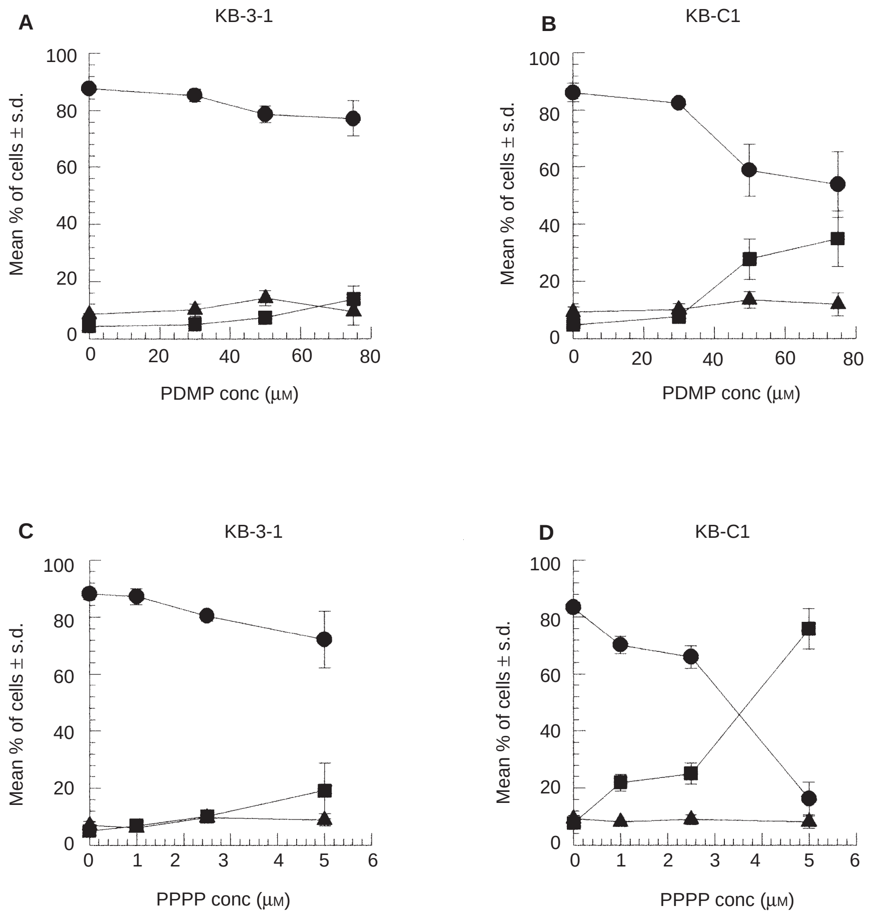

**Figure 2** The effect of exposure to PDMP or PPPP on KB cell lines. KB-3-1 cells were exposed to increasing concentrations of PDMP (**A**) or PPPP (**C**) and KB-C1 cells were exposed to PDMP (**B**) or PPPP (**D**) for 24 h. The percentage of viable (●), apoptotic (■) and necrotic cells (▲) was determined by assessing nuclear morphology, membrane integrity and phosphatidylserine externalization using Hoechst 33342, propidium iodide and annexin-V–FITC. Each point represents the mean of triplicate values from three independent experiments ± standard deviation the nucleus appeared fragmented and condensed. Late apoptotic cells were similar in appearance to early apoptotic cells although they appeared pink since membrane integrity is lost as a late feature of apoptosis and thus the cells were able to take up propidium iodide. Necrotic cells were also pink in appearance, the cells were often enlarged and the nucleus was diffuse and intact. Viewing cells under a blue filter for annexin-V–FITC binding demonstrated that only the apoptotic cells had a visible green plasma membrane indicative of annexin-V–FITC binding to externalized phosphatidylserine. Externalization of phosphatidylserine is an early apoptotic event, which precedes nuclear changes (Martin et al, 1995). Viable and necrotic cells had no annexin-V–FITC staining present. (See Bell et al (1998) for photographic examples of each cell type.) A t-test was used to determine significance between values for MDR and sensitive cells.

### Western blot analysis of poly-(ADP-ribose)-polymerase (PARP) cleavage in cells exposed to PDMP or PPPP

Cells were seeded in 25-cm$^2$ flasks at a density of $2 \times 10^5$ cells and were incubated for 2 days at 37°C. DMEM was removed and replaced with 5 μM PPPP or 75 μM PDMP for various times of exposure. Cell lysates were prepared in sample buffer (30 mM Tris–HCl pH 7.8, 9% sodium dodecyl sulphate (SDS), 0.1 mM PMSF) and protein was determined using a BCA-protein assay (Pierce). SDS polyacrylamide gel electrophoresis (SDS-PAGE) and Western blotting was performed by loading 30 μg protein per sample on to a 10% polyacrylamide gel using a standard procedure (Laemmli et al, 1970). The primary antibody (rabbit anti-PARP, Boehringer Mannheim) was diluted 1:2000 and the secondary anti-rabbit IgG horseradish-peroxidase antibody was diluted 1:5000. Bands corresponding to intact PARP and its cleavage product (113 and 89 kDa respectively) were detected by enhanced chemiluminescence (ECL kit, Amersham).

### Lipid extraction and thin layer chromatography

Total cellular lipids were extracted from $2 \times 10^7$ cells with chloroform/methanol (2:1 by volume). After sample separation into two phases, the lower organic phase containing the lipids was collected and a second extraction was performed with a synthetic aqueous phase (chloroform/methanol/0.1 M potassium chloride, 6:47:47 by volume). The lower organic phase was collected and evaporated to dryness and the resulting lipids were resuspended in chloroform. Lipids were applied to silica gel 60 TLC plates (Merck) on an equal total lipid basis and were run in a solvent system consisting of chloroform/methanol/ammonium hydroxide (70:20:4 by volume) against standards of ceramide (Sigma) and glucosylceramide

© 1999 Cancer Research Campaign 

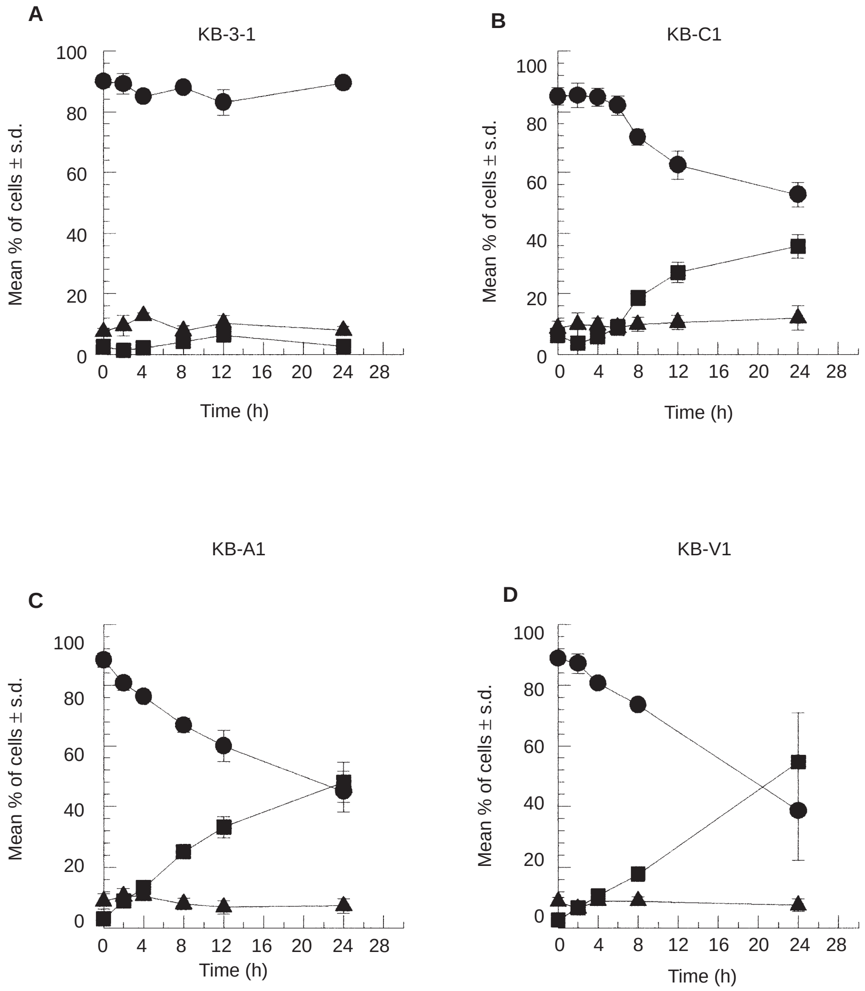

**Figure 3** A time course of apoptosis induction by 75 μM PDMP in (**A**) KB-3-1, (**B**) KB-C1, (**C**) KB-A1 and (**D**) KB-V1 cells as assessed by nuclear morphology, phosphatidylserine externalization and membrane integrity using Hoechst 33342, propidium iodide and annexin-V–FITC. Cells were scored as viable (●), apoptotic (■) or necrotic (▲). Each point represents the mean of triplicate values from three independent experiments ± standard deviation

(Sigma). Plates were air dried for 1 h before spraying with 35% sulphuric acid in water. Lipids were visualized by charring the plate at 160°C for 20 min.

### RESULTS

### Induction of apoptosis in MDR cell lines by PDMP and PPPP

Initial studies were performed to assess the effects of different concentrations of PDMP and PPPP on the levels of apoptosis and necrosis in normal and MDR cells. KB-3-1 and KB-C1 cells were plated in PDMP (30, 50 or 75 μM) or PPPP (1, 2.5 or 5 μM) for 24 h before the cells were harvested and labelled with Hoechst 33342, propidium iodide and annexin-V–FITC. Cells were scored as viable, apoptotic and necrotic on the basis of their nuclear morphology, membrane integrity and phosphatidylserine externalization. Figure 2 shows the effect of increasing inhibitor concentration on each cell line. The basal levels of apoptosis and necrosis in both cell lines were approximately 5% and 10% respectively. Increasing the concentration of PDMP in KB-3-1 cells increased the level of apoptosis only slightly and had no significant effect on the levels of necrosis. In contrast, increasing the concentration of PDMP in the KB-C1 cell line resulted in a marked dose-dependent increase in apoptosis to a maximum in 75 μM. Similarly, all concentrations of PPPP induced much higher levels of apoptosis in KB-C1 than in KB-3-1. On the basis of these results, it was decided to use concentrations of 75 μM PDMP and 5 μM PPPP in subsequent studies.

MDR cell lines selected in different drugs are known to have differences in cellular properties, including differing cross-resistance patterns (see, for example, Table 1 in Bell et al (1998)). Therefore, in order to broaden the significance of this work, all subsequent experiments were performed with the MDR cell lines KB-A1 and KB-V1, in addition to KB-C1. Initially, the effects of 24-h exposure to 75 μM PDMP or 5 μM PPPP on the cell survival of each of these three MDR cell lines was compared to that of the sensitive cell line KB-3-1. At the end of the 24-h exposure to the inhibitors, cell survival was determined by a colony-forming assay, performed in drug-free medium. In all cases, the inhibitors produced a much greater reduction in colony-forming ability in the MDR cell lines than in the sensitive cell line (Table 1). The effect

 © 1999 Cancer Research Campaign

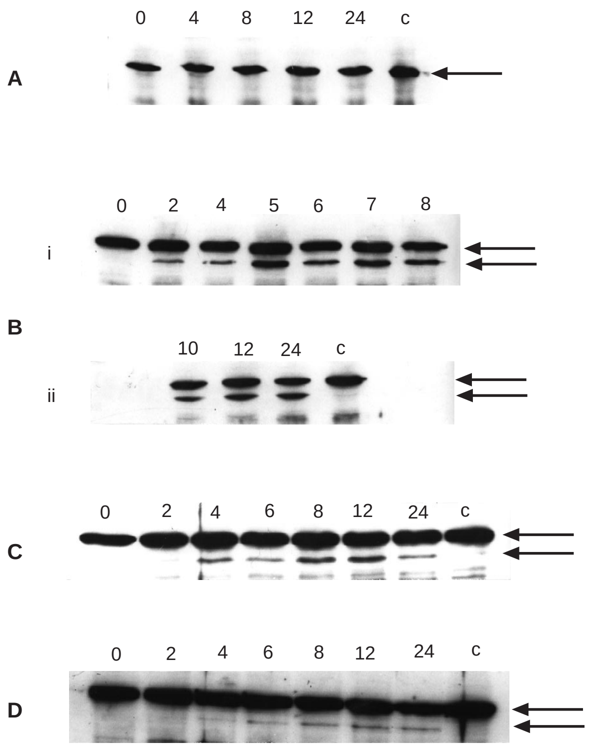

**Figure 4** Western blot of the cleavage of the enzyme poly-(ADP-ribose)-polymerase by PDMP. KB-3-1 (**A**), KB-C1 (**Bi** and **Bii**), KB-A1 (**C**) and KB-V1 (**D**) cells were exposed to 75 μM PDMP for up to 24 h. Numbers above lanes represent time of exposure (h) to PDMP. Untreated control cells (c) were harvested at 24 h. Arrows indicate intact PARP (113 kDa) and the cleavage product (89 kDa)

**Table 1** Percentage survival of KB-3–1, KB-C1, KB-A1 and KB-V1 cells following a 24-h exposure to 75 μM PDMP or 5 μM PPPP

| Cell line | Mean % survival ± s.d. after PDMP treatment | Mean % survival ± s.d. after PPPP treatment |
|---|---|---|
| KB-3-1 | 67.7 ± 6.2 | 63.4 ± 1.0 |
| KB-C1 | 31.9 ± 9.4[b] | 10.5 ± 2.5[c] |
| KB-A1 | 42.7 ± 2.5[b] | 1.7 ± 1.2[c] |
| KB-V1 | 36.2 ± 10.8[a] | 17.6 ± 0.6[c] |

Survival was determined using a colony formation assay by re-plating of the cells in drug-free medium for 8 days. Colonies of more than 50 cells were counted and the effect of PDMP or PPPP on colony forming ability compared to drug-free controls. Level of significance of the difference of the value for each MDR cell line is given in comparison to that for KB-3-1 ([a]$P < 0.05$, [b]$P < 0.01$, [c]$P < 0.001$).

was much more pronounced following treatment with 5 μM PPPP than with 75 μM PDMP. The greatest effect was on KB-A1, where treatment with PPPP for 24 h reduced cell survival to below 2%.

The levels of apoptosis and necrosis were then determined at various times of exposure to 75 μM PDMP up to 24 h in KB-3-1 and the three MDR KB cell lines. In KB-3-1 cells apoptosis and necrosis did not appear to increase over time (Figure 3). In all the MDR cell lines, apoptosis increased steadily over time concomitant with a reduction in the proportion of viable cells beginning after approximately 4 h of drug exposure. In all three MDR cell lines necrosis was not significantly altered over time of exposure. Maximum levels of apoptosis obtained following a 24-h exposure to PDMP were 34.8 ± 9.7% for KB-C1, 43.5 ± 16.9% for KB-A1 and 54.6 ± 16.3% for KB-V1 cells (Table 2) indicating that of the three MDR cell lines tested, KB-V1 cells were most susceptible to the effects of PDMP.

Cells were exposed to 5 μM PPPP for 24 h and the levels of viable, apoptotic and necrotic cells are presented in Table 3. Levels of apoptosis were significantly higher in the MDR cells than in KB-3-1 cells with values of 75.8 ± 7.1% for KB-C1, 73.6 ± 9.8% for KB-A1 and 75.3 ± 6.4% for KB-V1 cells obtained. In contrast, necrosis did not increase in relation to control values in any cell line. These results demonstrate that all three MDR cell lines exhibited similar high levels of sensitivity to PPPP and were more susceptible to the effects of PPPP than PDMP.

### PARP cleavage following exposure to PDMP or PPPP

The cleavage of the enzyme PARP was assessed as a second measure of apoptosis in addition to the cytological studies. PARP is cleaved in some cell lines by interleukin converting enzyme-like proteases as an early event in the induction of apoptosis from a 113 kDa protein into two fragments of 89 kDa and 24 kDa (Kaufmann et al, 1993). Cell lysates were prepared following a timed exposure to either inhibitor up to 24 h and the cleavage of PARP associated with the appearance of the 89 kDa cleavage product assessed by Western blotting. In KB-3-1 cells exposed to PDMP, no cleavage was detected after the maximum exposure of 24 h as determined by the absence of the 89 kDa band (Figure 4). In KB-C1 cells PARP was cleaved following a 2 h exposure to PDMP. In KB-A1 and KB-V1 cells exposed to PDMP, PARP was cleaved following a 4 h exposure. Cells exposed to PPPP and analysed for PARP cleavage are shown in Figure 5. As with PDMP, KB-3-1 cells exposed to PPPP for up to 24 h did not exhibit any PARP cleavage. In contrast, the three MDR cell lines studied showed PARP cleavage following exposure to PPPP for 2 h (KB-V1) or 8 h (KB-C1 and KB-A1). It is interesting to note that in the KB-V1 cell line only the cleavage fragment remained after a 24-h exposure to PPPP.

### Lipid analysis following exposure to PDMP and PPPP

Total cell lipids were extracted as described and were run on silica 60 thin layer chromatography (TLC) plates to resolve the different lipids. From Figure 6 it is clear that glucosylceramide was a minor lipid component of KB cells; although it was present at slightly higher levels in KB-A1 and KB-V1, an increase was not observed in KB-C1 cells in comparison to KB-3-1. Ceramide levels appeared to be similar in all four cell lines, although the possibility of other lipids co-migrating with ceramide has not been eliminated. Figure 7 shows the results produced following TLC of whole cell lipids in control and PDMP-treated cells. The level of glucosylceramide was reduced in each cell line by PDMP. This occurred concomitantly with a slight increase in ceramide levels in all cell lines except KB-A1. The most consistent and striking difference on treatment with PDMP related to an increase in a different lipid component which at present remains unidentified, but from studies using other lipid standards it has been demonstrated that this lipid was not sphingomyelin, sphingosine or sphinganine (data not shown).

The effect of the more specific inhibitor, PPPP on cellular lipids in KB cells is shown in Figure 8. PPPP treatment resulted in the absence of glucosylceramide in all the cell lines. An increase in ceramide levels was not detected in any cell line following treatment

© 1999 Cancer Research Campaign

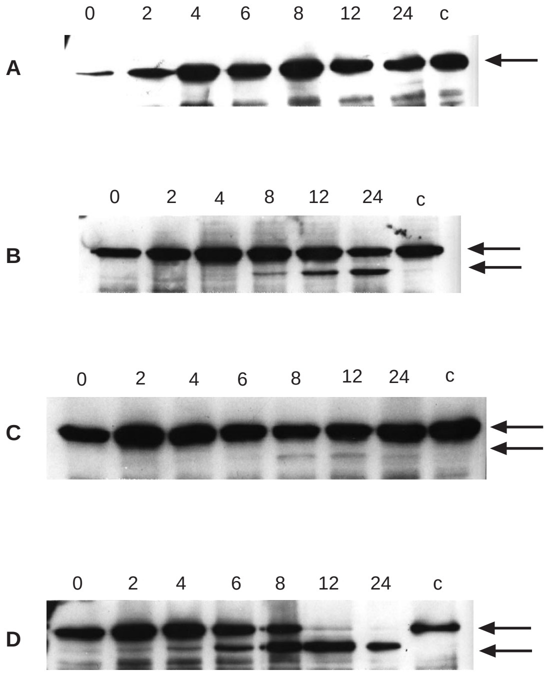

**Figure 5** Western blot of the cleavage of the enzyme poly-(ADP-ribose)-polymerase by PPPP. KB-3-1 (**A**), KB-C1 (**B**), KB-A1 (**C**) and KB-V1 (D) cells were exposed to 5 μM PPPP for up to 24 h. Numbers above lanes represent time of exposure (h) to PPPP. Untreated control cells (c) were harvested at 24 h. Arrows indicate intact PARP (113 kDa) and the cleavage product (89 kDa)

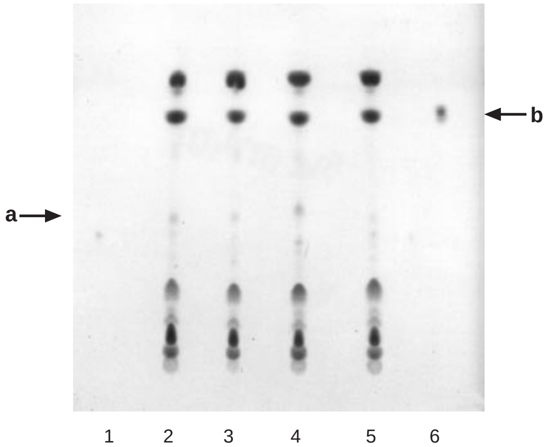

**Figure 6** Thin layer chromatogram of total cell lipids in untreated KB-3-1 (lane 2), KB-C1 (lane 3), KB-A1 (lane 4) and KB-V1 (lane 5) cells against a glucosylceramide standard (lane 1) and a ceramide standard (lane 6). Equal lipids were applied to the TLC plate and the lipids resolved in a solvent system consisting of chloroform/methanol/ammonium hydroxide (70:20:4 by volume). Glucosylceramide (**a**) and ceramide (**b**) are indicated by arrows with PPPP. Exposure to PPPP had a much less pronounced effect on the levels of the unidentified lipid which had been observed following PDMP treatment.

### DISCUSSION

This study has investigated the preferential killing of three KB MDR cell lines following exposure to two inhibitors of ceramide metabolism, PDMP and PPPP. It has been shown that there is a substantially greater killing of all the three MDR cell lines than in sensitive cells by exposure to either of the inhibitors, and that this preferential killing of MDR cells is due to the greater susceptibility to induction of apoptosis in these MDR lines. In accord with this finding, both drugs induce much greater PARP cleavage in the MDR cell lines than in KB-3-1. The three MDR cell lines had previously been shown to undergo apoptosis much more readily than sensitive cells when exposed to 2-deoxy-D-glucose (Bell et al, 1998). The present study, with a completely different class of agent, now suggests that the MDR cells may be preferentially sensitive to the induction of apoptosis by a wide range of stimuli.

We have shown that the effects of PDMP and PPPP on ceramide metabolism in all of our cell lines are broadly as would be expected from published observations in other cell systems. It has been previously reported that, although both are inhibitors of glucosylceramide synthase, PDMP results in a decrease in glucosylceamide levels and an increase in ceramide levels, whereas PPPP also results in a decrease in glucosylceramide levels, without a detectable increase in ceramide levels (Abe et al, 1995). The difference between the effects of the two inhibitors is thought to be due to PDMP having a less specific effect on a number of other enzymes involved in ceramide metabolism. In the present work, we have observed that both of the inhibitors reduced glucosylceramide levels in all the cell lines. PDMP treatment resulted in a detectable increase in ceramide levels in several and a marked increase in an

**Table 2** Induction of apoptosis in KB cells following exposure to PDMP

| Cell line | Mean % of cells ± s.d. | | |
|---|---|---|---|
| | Viable | Apoptotic | Necrotic |
| KB-3-1 | 77.1 ± 6.2 | 13.7 ± 4.7 | 9.2 ± 4.7 |
| KB-C1 | 53.8 ± 11.6[a] | 34.8 ± 9.7[a] | 11.7 ± 4.1 |
| KB-A1 | 49.5 ± 16.2[a] | 43.5 ± 16.9[a] | 7.0 ± 2.4 |
| KB-V1 | 38.5 ± 16.1[a] | 54.6 ± 16.3[a] | 7.5 ± 2.0 |

Cells were exposed to 75 μM PDMP for 24 h before harvesting and staining with propidium iodide, Hoescht 33342 and annexin-V-FITC. The percentage of viable, apoptotic and necrotic cells in each sample were assessed in triplicate. Results were expressed as a mean percentage of cells ± s.d. of three independent experiments. Significance of the value for MDR cells is given in relation to KB-3-1 cells ([a]$P < 0.001$).

**Table 3** Induction of apoptosis in KB cells following exposure to PPPP

| Cell line | Mean % of cells ± s.d. | | |
|---|---|---|---|
| | Viable | Apoptotic | Necrotic |
| KB-3-1 | 72.1 ± 10.0 | 19.0 ± 9.8 | 8.8 ± 2.2 |
| KB-C1 | 16.2 ± 5.8[a] | 75.8 ± 7.1[a] | 8.0 ± 2.1 |
| KB-A1 | 20.4 ± 9.9[a] | 73.6 ± 9.8[a] | 6.1 ± 1.8 |
| KB-V1 | 18.8 ± 5.0[a] | 75.3 ± 6.4[a] | 6.0 ± 2.3 |

Cells were exposed to 5 μM PPPP for 24 h before harvesting and staining with propidium iodide, Hoescht 33342 and annexin-V–FITC. The percentage of viable, apoptotic and necrotic cells in each sample were assessed in triplicate. Results were expressed as a mean percentage of cells ± s.d. of three independent experiments. Significance of the value of MDR cells is given in relation to KB-3-1 cells ([a]$P < 0.001$).

*© 1999 Cancer Research Campaign*

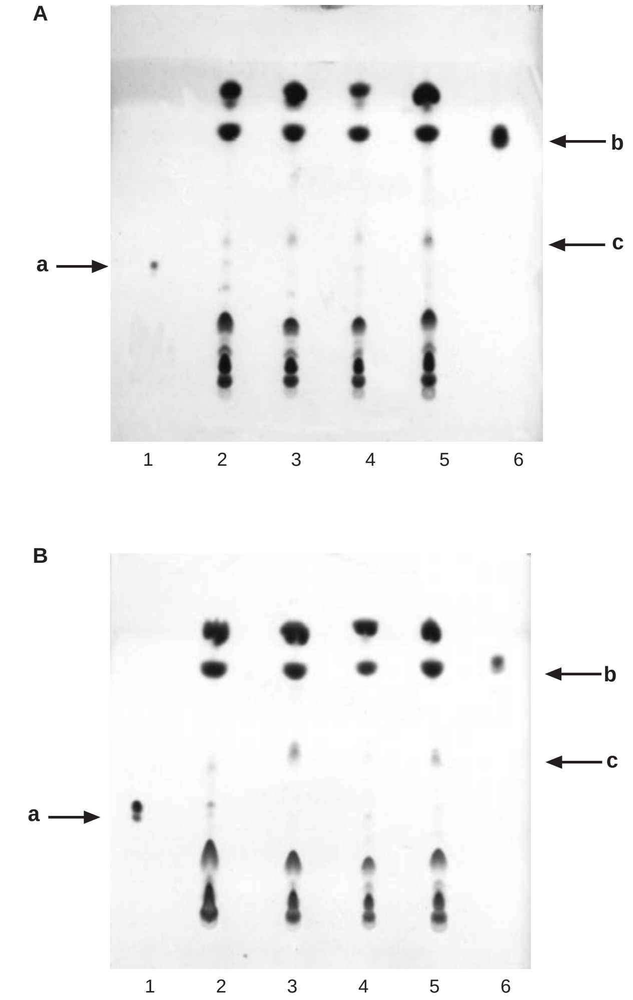

**Figure 7** (**A**) Thin layer chromatogram of total cell lipids in untreated KB-3-1 (lane 2) and untreated KB-C1 cells (lane 4). Lanes 3 and 5 represent total cell lipids from KB-3-1 and KB-C1 cells respectively following exposure to 75 μM PDMP for 24 h. (**B**) Thin layer chromatogram of total cell lipids in untreated KB-A1 (lane 2) and untreated KB-V1 cells (lane 4). Lanes 3 and 5 represent total cell lipids from KB-A1 and KB-V1 cells respectively following exposure to 75 μM PDMP for 24 h. Samples were run against a glucosylceramide standard (lane 1) and a ceramide standard (lane 6). Equal lipids were applied to the TLC plate and the lipids resolved in a solvent system consisting of chloroform/methanol/ammonium hydroxide (70:20:4 by volume). Glucosylceramide (**a**), ceramide (**b**) and the unknown lipid (**c**) are indicated by arrows

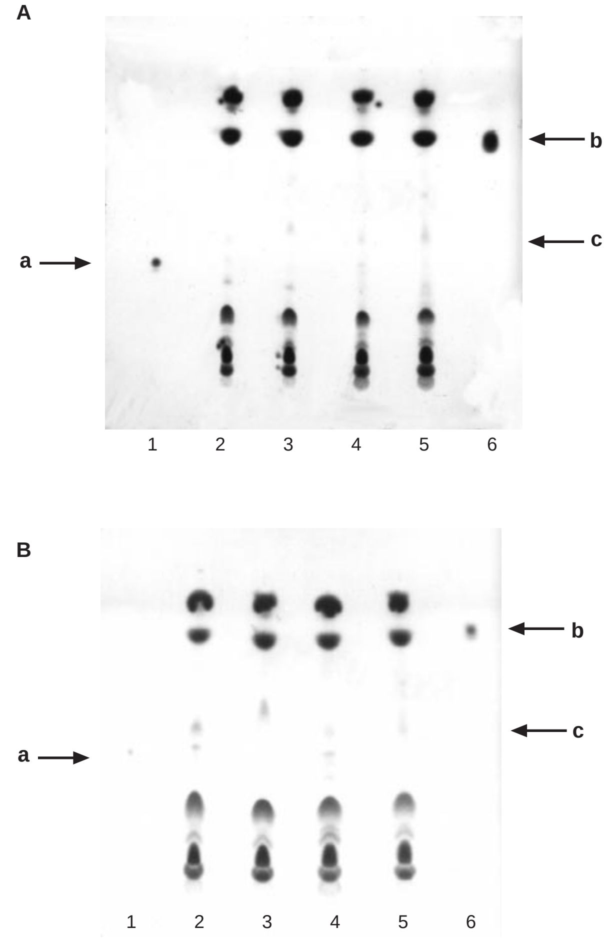

**Figure 8** (**A**) Thin layer chromatogram of total cell lipids in untreated KB-3-1 (lane 2) and untreated KB-C1 cells (lane 4). Lanes 3 and 5 represent total cell lipids from KB-3-1 and KB-C1 cells respectively following exposure to 5 μM PPPP for 24 h. (**B**) Thin layer chromatogram of total cell lipids in untreated KB-A1 (lane 2) and untreated KB-V1 cells (lane 4). Lanes 3 and 5 represent total cell lipids from KB-A1 and KB-V1 cells respectively following exposure to 5 μM PPPP for 24 h. Samples were run against a glucosylceramide standard (lane 1) and a ceramide standard (lane 6). Equal lipids were applied to the TLC plate and the lipids resolved in a solvent system consisting of chloroform/methanol/ammonium hydroxide (70:20:4 by volume). Glucosylceramide (**a**), ceramide (**b**) and the unknown lipid (**c**) are indicated by arrows unidentified lipid in all cell lines. In contrast, exposure to PPPP did not produce a detectable effect on ceramide levels in any of the cell lines, and had a much lesser effect than PDMP on the level of the unidentified lipid. Thus, as would be expected, PPPP has had a much more specific effect on glucosylceramide levels than PDMP.

The reduction in glucosylceramide level following inhibitor treatment occurred in KB-3-1 cells as well as in the MDR cell lines. Our results therefore suggest that maintenance of glucosylceramide levels are of much greater importance in preventing apoptosis in MDR cells than in their sensitive counterparts. This implies that the higher level of apoptosis, which is finally observed in response to the inhibitors in the MDR cells is due to a greater downstream susceptibility to changes in glucosylceramide levels in these cells. Glycosphingolipids such as glucosylceramide are known to have a role in cell growth and proliferation (Hannun and Bell, 1989; Rani et al, 1995), although their role in apoptosis is poorly defined. There are clear differences in the induction of PARP cleavage in all our MDR cell lines compared to KB-3-1, suggesting a difference in response in the MDR cells lies between glucosylceramide levels and PARP cleavage.

It is of interest that Lavie et al (1996) have previously reported elevated levels of glucosylceramide in three MDR cell lines (MCF-7-ADR, KB-V1 and OVCAR-3) in comparison to the levels in their sensitive parental cells and Lucci et al (1998) have suggested that elevated levels of glucosylceramide may be a convenient general marker by which to recognize MDR clinical samples. Our finding that glucosylceramide levels are increased in KB-V1 and KB-A1 is consistent with Lavie's observation, although the absence of such an increase in KB-C1 suggests that elevation of glucosylceramide levels may not be a universal feature of all MDR cell lines. (However, KB-C1 was selected in the presence of colchicine and therefore, overall, the data is still

*© 1999 Cancer Research Campaign*

consistent with the view that MDR cells which have been selected in the presence of those agents, which are clinically relevant do appear to have elevated glucosylceramide.) If, as we are suggesting here, that MDR cells are more sensitive to depletion of glucosylceramide than their non-MDR counterparts, observed increases in glucosylceramide in MDR cells may well have been selected as a buffering mechanism against fluctuations in glucosylceramide during cell growth or exposure to stress.

Our findings suggest that manipulation of glucosylceramide levels may be a fruitful way of causing the preferential killing of MDR cells. Further studies on the biochemical basis of the greater susceptibility to reduction of glucosylceramide in MDR cells may offer further insight into novel approaches to maximizing their preferential killing in vitro and possibly in vivo. It may be of particular interest to examine the combined effects of glucosylceramide synthase inhibitors and other cytotoxic agents on the preferential killing of MDR cells.

### ACKNOWLEDGEMENTS

This work was supported by a grant from Yorkshire Cancer Research. The authors wish to thank Ann Bamford for excellent technical help and Martin Rumsby for advice on TLC.

### REFERENCES

Abe A, Radin NS, Shayman JA, Wotring LL, Zipkin RE, Sivakumar R, Ruggieri JM, Carson KG and Ganem B (1995) Structural and stereochemical studies of potent inhibitors of glucosylceramide synthase and tumor cell growth. *J Lipid Res* **36**: 611–621

Bell SE, Quinn DM, Kellett GL and Warr JR (1998) 2-deoxy-D-glucose preferentially kills multidrug-resistant human KB carcinoma cell lines by apoptosis. *Br J Cancer* **78**: 1464–1470

Bentley J, Bell SE, Quinn DM, Kellett GL and Warr JR (1996) 2-deoxy-D-glucose toxicity and transport in human multidrug-resistant KB carcinoma cell lines. *Oncol Res* **8**: 77–84

Bentley J, Quinn DM, Pitman RS, Warr JR and Kellett GL (1997) The human KB multidrug-resistant cell line KB-C1 is hypersensitive to inhibitors of glycosylation. *Cancer Lett* **115**: 221–227

Cohen JS and Lyon RC (1987) Multinuclear NMR study of the metabolism of drug-sensitive and drug-resistant human breast cancer cells. *Ann NY Acad Sci* **508**: 216–228

Dobrowsky RT and Hannun YA (1992) Ceramide stimulates a cytosolic protein phosphatase. *J Biol Chem* **267**: 5048–5051

Hannun YA and Bell RM (1989) Functions of sphingolipids and sphingolipid breakdown products in cellular regulation. *Science* **243**: 500–507

Hoffman K and Dixit VM (1998) Ceramide in apoptosis – does it really matter? *TIBS* **23**: 374–377

Jarvis WD, Grant S and Kolesnick RN (1996) Ceramide and the induction of apoptosis. *Clin Cancer Res* **2**: 1–6

Kaufmann SH, Desnoyers S, Ottaviano Y, Davidson NE and Poirier GG (1993) Specific cleavage of poly-(ADP-ribose)-polymerase: an early marker of chemotherapy-induced apoptosis. *Cancer Res* **53**: 3976–3985

Laemmli UK (1970) Cleavage of structural proteins during the assembly of the head of bacteriophage T4. *Nature* **227**: 680–685

Lavie Y, Cao H, Bursten SL, Giuliano AE and Cabot MC (1996) Accumulation of glucosylceramides in multidrug-resistant cells. *J Biol Chem* **271**: 19530–19536

Ling V (1992) P-glycoprotein and resistance to anti-cancer drugs. *Cancer* **69**: 2603–2609

Lozano J, Berra E, Municio MM, Diaz-Meco MF, Dominguez I, Sanz L and Moscat J (1994) Protein kinase C-ζ is critical for NFκB-dependent promoter activation by sphingomyelinase. *J Biol Chem* **269**: 19200–19202

Luberto C and Hannun YA (1998) Sphingomyelin synthase, a potential regulator of intracellular levels of ceramide and diacylglycerol during SV 40 transformation. Does sphingomyelin synthase account for the putative phosphatidylcholine-specific phospholipase C? *J Biol Chem* **273**: 14550–14559

Lucci A, Cho WI, Han T, Giuliano AE, Morton DL and Cabot MC (1998) Glucosylceramide: a marker for multiple-drug resistant cancers. *Anticancer Res* **18**: 475–480

Marsh NL, Elias PM and Holleran WM (1995) Glucosylceramides stimulate murine epidermal hyperproliferation. *J Clin Invest* **95**: 2903–2909

Martin SJ, Reutelingsperger CPM, McGahon AJ, Rader JA, van Schie RCAA, LaFace DM and Green DR (1995) Early redistribution of plasma membrane phosphatidylserine is a general feature of apoptosis regardless of the initiating stimulus: inhibition by overexpression of Bcl-2 and Abl. *J Exp Med* **182**: 1545–1556

Mathias S, Dressler KA and Kolesnick RN (1991) Characterization of a ceramide-activated protein kinase: stimulation by tumor necrosis factor-α. *Proc Natl Acad Sci USA* **88**: 10009–10013

Radin NS (1994) Rationales for cancer chemotherapy with PDMP, a specific inhibitor of glucosylceramide synthase. *Mol Chem Neuropathol* **21**: 111–127

Radin NS (1996) Treatment of Gaucher disease with an enzyme inhibitor. *Glycoconj J* **13**: 153–157

Radin NS, Shayman JA and Inokuchi JI (1993) Metabolic effects of inhibiting glucosylceramide synthesis with PDMP and other substances. *Adv Lipid Res* **26**: 183–213

Rani CS, Abe A, Chang Y, Rosenzweig N, Saltiel AR, Radin NS and Shayman JA (1995) Cell cycle arrest induced by an inhibitor of glucosylceramide synthase. *J Biol Chem* **270**: 2859–2867

Volm M (1998) Multidrug resistance and its reversal. *Anticancer Res* **18**: 2905–2918

 © 1999 Cancer Research Campaign
